# Methylator phenotype of malignant germ cell tumours in children identifies strong candidates for chemotherapy resistance

**DOI:** 10.1038/bjc.2011.218

**Published:** 2011-06-28

**Authors:** J N Jeyapalan, D A Mohamed Noor, S-H Lee, C L Tan, V A Appleby, J P Kilday, R D Palmer, E C Schwalbe, S C Clifford, D A Walker, M J Murray, N Coleman, J C Nicholson, P J Scotting

**Affiliations:** 1 Children's Brain Tumour Research Centre, Centre for Genetics and Genomics, University of Nottingham, Queen's Medical Centre, Nottingham NG7 2UH, UK; 2 Children's Brain Tumour Research Centre, Child Health, School of Clinical Sciences, University of Nottingham, Queen's Medical Centre, Nottingham NG7 2UH, UK; 3 MRC Cancer Cell Unit, Hutchison/MRC Research Centre, Box 197, Hills Road, Cambridge CB2 0XZ, UK; 4 Northern Institute for Cancer Research, Sir James Spence Institute, Newcastle University, Newcastle-upon-Tyne NE2 4HH, UK; 5 Department of Paediatric Oncology, Addenbrooke's Hospital, Box 181, Hills Road, Cambridge CB2 0QQ, UK

**Keywords:** germ cell tumour, yolk sac tumour, germinoma, methylation, paediatric

## Abstract

**Background:**

Yolk sac tumours (YSTs) and germinomas are the two major pure histological subtypes of germ cell tumours. To date, the role of DNA methylation in the aetiology of this class of tumour has only been analysed in adult testicular forms and with respect to only a few genes.

**Methods:**

A bank of paediatric tumours was analysed for global methylation of LINE-1 repeat elements and global methylation of regulatory elements using GoldenGate methylation arrays.

**Results:**

Both germinomas and YSTs exhibited significant global hypomethylation of LINE-1 elements. However, in germinomas, methylation of gene regulatory regions differed little from control samples, whereas YSTs exhibited increased methylation at a large proportion of the loci tested, showing a ‘methylator’ phenotype, including silencing of genes associated with Caspase-8-dependent apoptosis. Furthermore, we found that the methylator phenotype of YSTs was coincident with higher levels of expression of the DNA methyltransferase, *DNA (cytosine-5)-methyltransferase 3B*, suggesting a mechanism underlying the phenotype.

**Conclusion:**

Epigenetic silencing of a large number of potential tumour suppressor genes in YSTs might explain why they exhibit a more aggressive natural history than germinomas and silencing of genes associated with Caspase-8-dependent cell death might explain the relative resistance of YSTs to conventional therapy.

Changes in DNA methylation have been thought to have a role in cancer aetiology for many years (Feinberg and Vogelstein, 1983; Feinberg and Tycko, 2004). Although hypomethylation of repetitive elements has been shown in a range of cancer types, there is little evidence for hypomethylation of gene regulatory sequences (De Smet and Loriot, 2010). By contrast, analysis of many individual tumour suppressor genes has shown their repression by promoter hypermethylation in cancers to be commonplace. More recently, where global analyses have been applied, all classes of tumour analysed have shown high levels of methylation of many genes, suggesting that this is a widespread event in cancer development (Martin-Subero *et al*, 2009; Richter *et al*, 2009). Indeed, specific subgroups of colon cancer and glioma have been shown to exhibit a ‘methylator’ phenotype (Toyota *et al*, 1999; Weisenberger *et al*, 2006; Shen *et al*, 2007; Noushmehr *et al*, 2010).

Children's cancers develop over a very short time scale, many within the first few years and even perinatally. Unlike cancers in adults, this would not seem to provide time for a series of carcinogenically driven mutations to arise. Dysregulation of gene expression due to changes in global methylation through a defect in the methylation machinery provides a plausible alternative mechanism for the development of these childhood cancers.

Paediatric germ cell tumours (GCTs) are a particularly unusual group of cancers. They arise not only in the tissues where germ cells would normally reside, the gonads, but also in extragonadal sites, primarily the base of the spine (sacrococcygeal tumours), the thorax (mediastinal tumours) and in the ventral midline of the brain. In addition, they exhibit strikingly different histological subtypes that parallel the forms seen in adults, classified as seminomatous or non-seminomatous. The seminomatous tumours are uniform tumours resembling those found in adult testes with a similarity to germ cell progenitors (in extragonadal sites, and throughout this report, referred to as ‘germinomas’). Non-seminomatous tumours represent several morphologically distinct subtypes, of which yolk sac tumours (YSTs) are the major class in children and which are therefore included in this study. In general, YSTs are relatively more aggressive and resistant to therapy than seminomatous tumours (Murray *et al*, 2010). Despite these differences, all GCTs are believed to share a common lineage, as both histological subtypes can exist in the same tumour (Looijenga, 2009) and primary seminomatous tumours can recur as non-seminomatous tumours (Wong *et al*, 2010).

Like other paediatric tumours, GCTs may exhibit relatively few cytogenetic abnormalities and the teratoma subtype, for example, exhibits little or no cytogenetic damage (Rickert, 1999; Mostert *et al*, 2000; Veltman *et al*, 2003; Oosterhuis and Looijenga, 2005). Hence, in cases where DNA damage is not the primary cause of dysregulated gene expression, then it must be disrupted by another mechanism, for which DNA methylation is a strong candidate. For this reason, analysing the methylation status of the genome is of particular importance to our understanding of this class of tumour.

To date, DNA methylation has only been analySed with respect to a small group of genes in adult testicular GCTs (Koul *et al*, 2002, 2004; Smith-Sorensen *et al*, 2002; Honorio *et al*, 2003; Manton *et al*, 2005; Lind *et al*, 2006, 2007), which revealed the methylation of nine known tumour suppressor genes in a large number of tumours. However, no analysis has been carried out to determine if changes in methylation are seen in paediatric cases or in extragonadal GCTs.

To analyse methylation in a cohort of paediatric GCTs, we adopted two approaches. Firstly, we analysed the methylation status of the LINE-1 repetitive elements that are dispersed throughout the genome and have been shown to reflect the general methylation status of intergenic DNA. Secondly, we also used GoldenGate methylation arrays (Illumina, San Diego, CA, USA) to assess the methylation status of more than 800 genes. These experiments revealed a striking difference in methylation between YSTs and germinomas, which occurred irrespective of anatomical location, sex or age. This makes YSTs a very unusual histological class of tumour in which the great majority exhibit a highly methylated state, setting them apart from other types of cancer in which DNA methylation has been analysed to date. Our study identified a large number of potentially important methylation events that could contribute to the tumour's pathogenesis, among which the methylation of several genes correlated well with their expression levels between the two groups of tumours. Most notable among these were genes associated with the extrinsic pathway of apoptosis. Finally, we found that the ‘methylator’ phenotype in YST correlated with increased expression of *DNA (cytosine-5)-methyltransferase 3B* (*DNMT3B*).

## Materials and methods

### Combination of bisulphite and restriction analysis

LINE1 PCR was carried out according to Yang *et al* (2004) using 30 ng of bisulphite converted (EZ DNA methylation kit, Zymo Research, Irvine, CA, USA) genomic DNA and Platinum *Taq* (Invitrogen, Paisley, UK), with 100 nM of FAM-labelled forward primer and 100 nM reverse primer. Polymerase chain reaction products were then digested with *Hinf*1 restriction enzyme (NEB, Hitchin, UK). Digested products were analysed by gel electrophoresis and GeneScan (Applied Biosystems, Foster City, CA, USA). Primers sequences are shown in Supplementary information and Supplementary Table 3.

### Methylation microarray analysis

Methylation array analysis was performed at the Wellcome Trust Centre for Human Genetics, University of Oxford, using the Illumina GoldenGate Cancer Panel I assay http://www.illumina.com/pages.ilmn?ID=193/, according to the manufacturer's instructions. The assay reports methylation values at 1505 loci mapping to 807 genes previously associated with DNA methylation and/or cancer.

Quality control was carried out using Beadstudio v.3.2 methylation module (Illumina) and the R package, *beadarray* (Dunning *et al*, 2007), which enabled the identification and exclusion of potentially confounding spatial artefacts. Samples failing quality control were removed from subsequent analyses. After background signal normalisation, the assay reported *β*-values for each measured probe, with values ranging from zero (unmethylated) to one (methylated) (Bibikova *et al*, 2006).

### Cluster analysis and identification of differentially methylated loci

Bootstrapped hierarchical clustering was performed using the R package *pvclust* (Suzuki and Shimodaira, 2006), using Euclidean distance, average agglomeration and 10 000 replications. Subgroups with an approximate unbiased *P*-value of <0.05 were significant. The observed clustering patterns were assessed using principal component analysis and *k* means analysis. The optimal number of clusters for *k* means analysis was assessed using Scree plots. Differentially methylated loci between subgroups were identified using Mann–Whitney *U*-tests, with a *P*-value<0.05 after Benjamini–Hochberg false discovery rate correction (Benjamini and Hochberg, 1995) for multiple testing, with an additional filter that the average change in *β* between subgroups be >0.2. This was increased to >0.34 for comparison between tumour subtypes.

### PCR and pyrosequencing

Polymerase chain reaction was carried out on bisulphite converted genomic DNA using Platinum *Taq* polymerase (Invitrogen). Polymerase chain reaction cycling conditions were 94°C for 10 min, followed by 45 cycles of 94°C for 60 s, 55°C for 60 s and 72°C for 40 s, with a final 5 min extension at 72^o^C. The biotin-labelled strand of the amplicon was isolated and pyrosequencing carried out using sequencing primers at the Genome Centre, Queen Mary University of London, London, UK. Primers sequences are shown in Supplementary information and Supplementary Table 3.

### Reverse transcription–PCR analysis

First-strand cDNA synthesis was performed using random primers (Promega, Southampton, UK) with SuperScript III Reverse Transcriptase (Invitrogen). Polymerase chain reaction was carried out using 0.04 U *μ*l^−1^ Kapa *Taq* (GRI, Essex, UK). Minus RT controls were routinely performed. Primers sequences are shown in Supplementary information and Supplementary Table 3.

## Results

### Global methylation of LINE-1 repetitive elements

**Figure 1:**
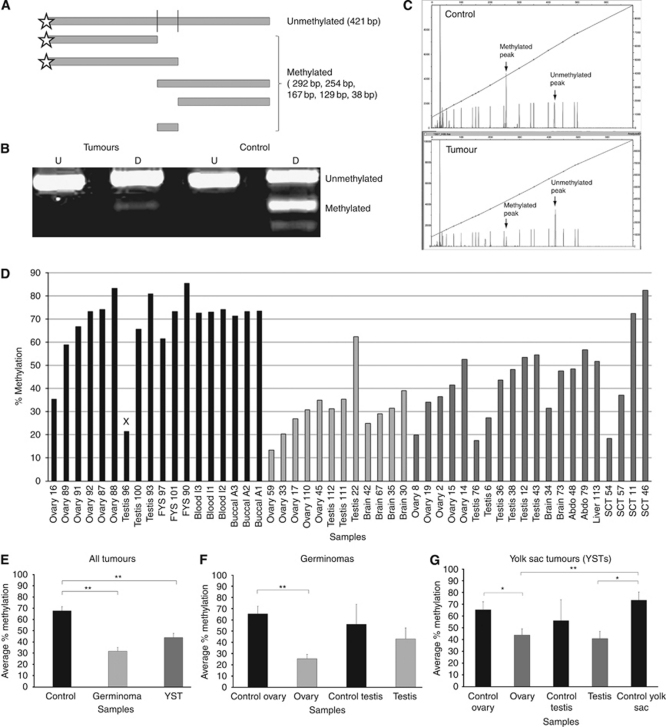
Combination of bisulphite and restriction analysis (COBRA) of LINE-1 element methylation. (**A**) Diagram showing predicted digestion products. (**B**) Illustration of undigested (U) and digested (D) PCR products from representative tumour and control samples. (**C**) Example of GeneScan data from a representative control and tumour samples. (**D**) Percentage methylation levels of LINE-1 elements from individual samples as determined by GeneScan quantification. (**E**–**G**) Comparisons between tumour and control groups of samples showing that LINE-1 elements are significantly hypomethylated in all tumour groups as compared with controls, except where testicular tumours were compared with normal testes samples. However, the latter appears to reflect one of the three testicular control samples having a very low methylation value (*x* in **D**). ^*^P<0.05, ^**^P<0.01.

We first examined the global methylation status of 32 tumours from a bank of paediatric GCTs (Supplementary information and Supplementary Table 1) using the well-established strategy of analysing the LINE-1 repetitive element sequences via the combination of bisulphite and restriction analysis (COBRA) technique (Yang *et al*, 2004; Figure 1A–C). These sequences are normally heavily methylated, which is believed to be important to maintain them in a ‘silent’ state so that they cannot destabilise the genome (Belancio *et al*, 2010).

Consistent with studies in other cancers, the level of methylation of the LINE-1 elements in almost all GCT samples analysed was lower than that in controls (Figure 1D and E). Control samples averaged 68% methylation, whereas the level of methylation was significantly lower (*P*<0.0001) in germinomas (32%) and in YSTs (42%). These differences were also seen between tumours and tissue-matched controls (Figure 1F and G).

### Global methylation analysis of gene regulatory sequences

**Figure 2:**
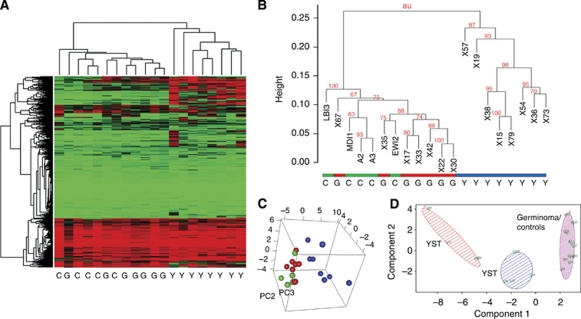
Cluster analysis of tumours according to methylation status. (**A**) Heat map showing that germinomas (G) cluster together with controls (C), whereas YSTs (Y) cluster separately, showing higher levels of methylation (red). (**B**) Bootstrapped hierarchical clustering using the R package *pvclust* (Suzuki and Shimodaira, 2006). Subgroups with an approximate unbiased *P*-values of <0.05 were significant. (**C**) The observed clustering patterns were assessed using principal component analysis and *k* means analysis. Yolk sac tumours are shown in blue, germinomas in red and controls in green. (**D**) Plot shows subgroup members selected by *k* means analysis plotted against the first two principal components. The optimal number of clusters for *k* means analysis was assessed using Scree plots. Differentially methylated loci between subgroups were identified using Mann–Whitney *U*-tests, with an adjusted *P-*value <0.05 after Benjamini–Hochberg false discovery rate correction for multiple testing. The colour reproduction of this figure is available at the *British Journal of Cancer* online.

We next used the GoldenGate beadchip system to assess the methylation status of gene regulatory regions. These beads carry an optimised set of 1505 CpG sites selected from 807 genes including known tumour suppressors, oncogenes and factors involved in processes such as DNA repair, cell cycle control, differentiation and apoptosis. Results were obtained for 15 tumours (seven germinomas and eight YSTs) and five control samples from healthy individuals (buccal cells from two adult male subjects and peripheral blood from three infants) (Supplementary Tables 1 and 2). Unsupervised hierarchical clustering grouped the germinomas and controls together, whereas YSTs formed an independent cluster exhibiting a generally more methylated status (Figure 2A and B). Using three-dimensional principle component analysis, YSTs again formed a separate group from the germinomas and controls, which formed a single group (Figure 2C). Even when we used *k*-means analysis of the 20 samples (a preceding Scree plot had determined that three clusters were optimal) germinomas remained clustered together with the control samples and it was the YSTs that were separated into two distinct groups (Figure 2D).

### YSTs but not germinomas exhibit widespread hypermethylation of gene regulatory sequences

To describe differences between *β*-values (delta *β*-value) that were considered ‘significant’, we analysed an additional, independent cohort of nine germinomas and 11 YSTs (Supplementary Tables 1 and 2). This revealed that the majority of delta *β*-values >0.3 were consistent between the two tumour subtypes in both cohorts of samples. We therefore used this delta *β*-value to determine significant differences in methylation. It is worth noting that this value is among the more stringent values used in previous studies (Martin-Subero *et al*, 2009; Richter *et al*, 2009; Ang *et al*, 2010).

Of the genes included on the GoldenGate array, 131 (16%) were significantly more methylated in YSTs than in germinomas (Supplementary Table 1; X-chromosome genes were excluded as they would normally differ between male and female subjects due to X-chromosome inactivation.). Importantly, we also determined which genes were hypermethylated or hypomethylated in GCTs as compared with ‘normal’ control tissues. Overall, we found little evidence of hypomethylation. In germinomas, only 4 out of 807 genes (0.5% of genes tested) were significantly less methylated than controls and only 13 out of 807 genes were hypomethylated in YSTs. Indeed, only one gene, *CD86*, was reproducibly hypermethylated in germinomas and there is no evidence to suggest that this cell surface marker can function as a tumour suppressor.

Therefore, comparison to normal control tissues allowed us to show that the germinomas in our study have a normal level of methylation of gene regulatory regions, not ‘as good as devoid of DNA methylation’ as was concluded by Lind *et al* (2007) with respect to seminomatous GCTs. Such a lack of hypermethylation makes germinomas a very unusual tumour type compared with the other classes of tumour analysed to date.

### Many potential tumour suppressor genes are methylated in YSTs

**Figure 3:**
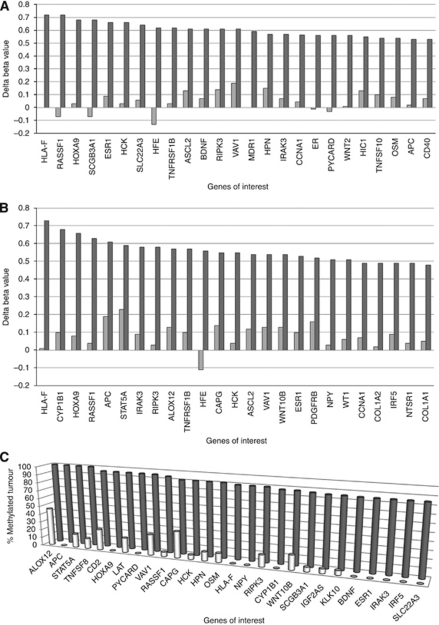
Graphical representation of the 25 most differentially methylated genes in YSTs. Bars represent average difference of methylation to controls for each gene in either YSTs (dark bars) or germinomas (pale bars) in the first (**A**) and second (**B**) cohorts of tumours analysed. (**C**) The percentage of tumours across both cohorts analysed in which each gene was hypermethylated in either YSTs (dark bars) or germinomas (pale bars).

In our initial array analysis, 85 out of 807 genes (10.5%) were hypermethylated in YSTs, but not germinomas (Supplementary Table 2; top 25 shown in Figure 3A). In this study, the genes identified to be specifically hypermethylated in paediatric YSTs were highly reproducible. In the second cohort of GCTs analysed, a similar number of genes were hypermethylated (104 out of 807, 12.8%), 71 of which were the same genes identified in the first cohort, with three genes appearing in the top five (as ranked by delta *β*-value) in both cohorts (Figure 3B). Indeed, analysis of each tumour for the methylation status of the 85 genes initially identified showed that 27 of these genes were hypermethylated in ⩾85% of tumours from both cohorts (Figure 3C). Hence, it appears that the significant majority of YSTs exhibit a very similar ‘methylator’ phenotype.

These hypermethylated genes included several that others have shown to be highly methylated in adult testicular non-seminomas. Previous studies used a candidate gene approach to identify nine genes, from a total of 39 studied, that were frequently methylated in non-seminomatous adult testicular GCTs, especially in YSTs (Koul *et al*, 2002, 2004; Smith-Sorensen *et al*, 2002; Honorio *et al*, 2003; Manton *et al*, 2005; Lind *et al*, 2006, 2007). Of these previously identified genes, we found that five (*RASSF1A*, *HOXA9*, *SCGB3A1*, *HIC1*, *APC*) of the nine included in our initial array analysis were also significantly more methylated in YSTs than in germinomas.

Previously, *RASSF1A*, *HOXA9*, *SCGB3A1*, *HIC1* and *APC* had been shown to be hypermethylated in 100% of YSTs analysed (Honorio *et al*, 2003; Rathi *et al*, 2003; Koul *et al*, 2004; Lind *et al*, 2006; Brieger *et al*, 2010). However, we found only one of these genes hypermethylated in all YST samples from both cohorts of tumours analysed (Figure 3C). This was largely due to the presence of a single YST in the second cohort that lacked the methylator phenotype (approximately 74% of the genes that were hypermethylated in most other YSTs exhibited no significant hypermethylation in this tumour). To gain a clear indication of those genes most strongly associated with the methylator phenotype, we next excluded the single YST lacking the methylator phenotype. We found three genes hypermethylated in all of these (*HOXA9*, *APC*, *PYCARD*), which were methylated in less than 25% of germinomas and a further 30 genes hypermethylated in over 80% of the YST samples (14 of these genes were not methylated in a single germinoma and 16 were methylated in <25% of germinomas; Table 1).

Among the genes selectively methylated in YSTs, many have been previously implicated as tumour suppressors (Table 2). However, rather surprisingly, of the ‘top 14’ most frequently reported genes that are hypermethylated in human cancers (Cheung *et al*, 2009), all of which were included in our array analysis, only *RASSF1A*, *APC* and *ESR1* were methylated in the majority of YSTs and almost none were methylated in germinomas. Even more striking is the fact that a recent analysis of gliomas exhibiting a ‘methylator’ phenotype showed 12 of these 14 genes to be hypermethylated, but *APC* and *ESR1* are the very genes that did not show hypermethylation in these gliomas (Noushmehr *et al*, 2010). Our findings therefore suggest that the vast majority of YSTs exhibit a strong methylator phenotype, but that this is quite a different methylation signature to that seen in other tumour types (Weisenberger *et al*, 2006; Shen *et al*, 2007), suggesting a different underlying mechanism.

### Validation of methylation array data

**Figure 4:**
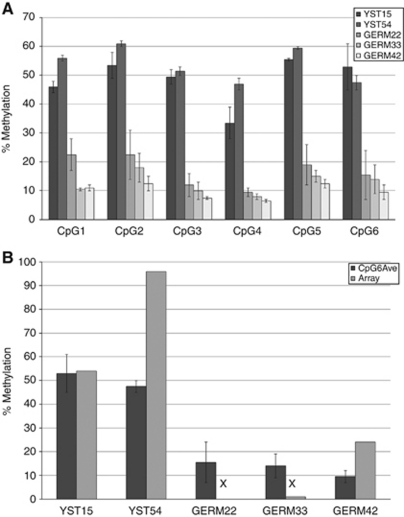
Pyrosequence of the CpG island of the *PYCARD* gene from selected tumour samples. (**A**) Graph showing percentage methylation of two YST and three germinoma samples at the six CpG positions included in the region of pyrosequencing for the *PYCARD* gene (100 bp between positions −234 and −135 proximal to the start of transcription). The YST samples show clear hypermethylation when compared with the germinoma samples. (**B**) Comparison between the percentage methylation at the sixth CpG (−151) in the five tumours shown in (**A**), as determined from either the pyrosequencing (dark bars) or from the methylation array (pale bars). This shows strong correlation between the array and pyrosequencing results and also suggests that the pyrosequencing gives more quantitative values at lower levels of methylation where these are below the level detectable in the array (arrow heads). Bars show the range for the two samples.

Pyrosequencing of bisulphite-treated DNA was used to validate the data from the GoldenGate arrays, which also extended the number of CpGs analysed. We selected *PYCARD* for this purpose as, although this gene was significantly hypermethylated in almost all YSTs and, as discussed below, this was reflected in a difference in its expression between YSTs and germinomas, the difference in the level of methylation between germinomas and YSTs was moderate. *PYCARD* therefore represented a stringent validation test of the methylation arrays. Owing to the limited availability of the tumour DNA samples, this analysis was carried out only on five samples in duplicate, which showed that the entire protocol was highly reproducible (Figure 4A).

Pyrosequencing was carried out on the proximal promoter region of *PYCARD* from a position −234 bp proximal to the transcription start site. The amplified sequence was 100 bp including six CpGs, one of which (at position −151) was also included in the arrays. Comparison of the methylation values obtained for this single CpG between pyrosequencing and the original arrays showed a strong correlation between the two techniques (Figure 4B). Pyrosequencing also revealed that CpGs adjacent to this CpG shared a similar methylation status (Figure 4A). These data validated the difference in methylation of *PYCARD* between germinomas and YSTs and showed that this difference was shared with other, nearby CpGs.

### Relationship between methylation and gene expression

If differences in gene methylation are of biological significance to the pathogenesis of GCTs, it seems probable that they must affect the expression of those genes. In the previous studies of methylated genes in adult testicular GCTs, only one gene, *RASSF1A*, which was methylated in a large proportion of non-seminomatous tumours and unmethylated in most seminomatous tumours, exhibited an expression pattern that correlated with its methylation status between the two tumour subtypes (Koul *et al*, 2002). To determine if the methylation we saw correlated with gene expression in the tumour cells, we compared our data to the expression array analysis previously reported for this same bank of tumours (Palmer *et al*, 2008). Overall, the majority of hypermethylated genes showed no significant difference in expression (taken as an LOD score or B-statistic value of 3 or more). This may not be surprising, as absence of methylation is not the only prerequisite for a gene to be expressed. However, among the genes that we identified as methylated in YSTs, but not in germinomas, eight were selectively expressed in germinomas, whereas only one was selectively expressed in YSTs (Table 3).

Among the genes methylated in YSTs and selectively expressed in germinomas, *TFAP2C*, *PYCARD*, *CD2*, *CASP8* (*Caspase 8*), *EVI2A* and *HLA-F* were very biased in both methylation and expression, with LOD scores for the difference in expression >4 (Palmer *et al*, 2008) and hypermethylation in >75% of YSTs and in 25% or fewer germinomas (except *TFAP2C*, which was only hypermethylated in 63% of YSTs, but in no germinomas) (Figure 3C). It is striking that this list does not include the genes previously implicated by methylation in GCTs, such as *HOX9A* or *RASSF1A*, but instead identifies six new genes as the best candidates for a role of DNA methylation in GCT biology. Of these, *CASP8* and *PYCARD* are of particular interest as they are known to be associated with apoptosis.

### Cause of hypermethylation in YSTs

In other cancers, changes in specific components of the epigenetic machinery have been shown to cause hypermethylation. EZH2 and SUZ12, components of the polycomb PRC2 complex, have been implicated in several types of cancer (Chen *et al*, 2010; Karanikolas *et al*, 2010; Martin-Perez *et al*, 2010). Also, overexpression of the DNA methyltransferase, *DNMT3B*, was identified in lung and breast cancer cell lines (Beaulieu *et al*, 2002) and depletion of *DNMT3B* expression in breast cancer cell line was shown to activate the methylated *RASSF1* (Wang *et al*, 2006); therefore, we analysed the expression of these factors by qRT-PCR.

**Figure 5:**
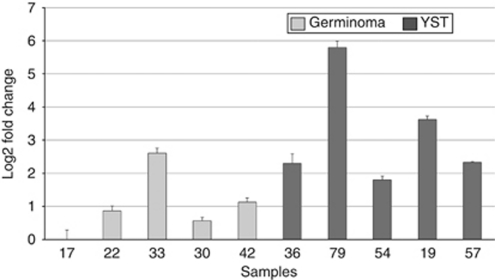
Quantitative RT–PCR analysis of *DNMT3B* expression. Graph showing the expression level of *DNMT3B* in five germinomas (pale bars) and five YST samples (dark bars). Error bars show standard deviation.

Although we had access to only a small number of RNA samples from the same tumours analysed for their methylation status, our data did reveal a significant difference between germinomas and YSTs in the level of expression of *DNMT3B* (Figure 5), but not *EZH2* or *SUZ12* (data not shown). *DNMT3B* was generally expressed at levels 4–16-fold greater in the YST samples analysed compared with the germinoma samples. Indeed, analysis of the array data on the same cohort of GCTs (Palmer *et al*, 2008) revealed that *DNMT3B* was more strongly expressed in YSTs than in germinomas (LOD scores of 3.36 and 6.7, respectively, *P*=0.0006). *DNMT3B* overexpression therefore provides a strong candidate to explain the methylator phenotype seen in those YSTs.

## Discussion

Previous studies have identified a small number of genes that are hypermethylated in adult testicular GCTs, with non-seminomatous tumours exhibiting much higher levels of methylation than their seminomatous counterparts. In this study, we have performed the first array-based analysis of the methylation status of GCT genomes. In particular, we have studied paediatric tumour samples from a range of gonadal and extragonadal locations. This has revealed that, while there is global hypomethylation of repetitive elements in both germinomas and YSTs and in GCTs from all anatomical locations, there is little hypomethylation of gene regulatory sequences. Germinomas also show little hypermethylation, whereas many genes are hypermethylated in YSTs regardless of tumours site, patient age or sex. The set of genes hypermethylated in YSTs was very consistent across two independent cohorts of tumour samples and so identifies a large number of candidate tumour suppressors for future functional analysis.

### The role of methylation in GCTs

Our study agrees with the recent consensus that reduced methylation of repetitive elements is not reflected in a general lack of methylation of gene regulatory elements (De Smet and Loriot, 2010). Hence, any contribution of global hypomethylation is likely to be through its effects on repeat elements. Two general models have been proposed. In the first, lack of methylation of repeat elements might mobilise them, resulting in genomic instability (Schulz *et al*, 2006), as was seen in a mouse model in which global methylation was disrupted owing to a hypomorphic mutation in *Dnmt1* (Gaudet *et al*, 2003; Howard *et al*, 2008). In the second model, activation of transcription of these elements might be associated with an increase in transcription of adjacent genes (Cruickshanks and Tufarelli, 2009; Weber *et al*, 2010). Given the general hypomethylation of LINE-1 elements in GCTs, the potential mechanism by which this may affect tumour development is worthy of further investigation.

As 131 of 807 genes analysed were hypermethylated in YSTs, it seems premature to assume that any one of these genes might have a significant role in the tumours’ aetiology. Indeed, earlier studies have not generally shown whether differences in methylation in GCTs correlated with any difference in the expression of those genes (Lind *et al*, 2007). It is clear from our data that the majority of the genes that are differentially methylated between germinomas and YSTs are not differentially expressed (Palmer *et al*, 2008). This provides a much smaller number of genes in which methylation status correlates with expression levels, which are therefore more likely to have a role in GCT pathogenesis. Although we found methylation of five of the previously identified genes that were known to be hypermethylated in YSTs, none of these exhibited a significant difference in expression between the two tumour subtypes.

Among the six genes where methylation state corresponded with expression, *CASP8* and *PYCARD* were of particular note as they both act in the same apoptotic pathway. Caspase 8 is critical for death receptor-induced, extrinsic apoptosis (triggered by the death ligand TRAIL/TNFSF10 and other tumour necrosis superfamily factors (TNFSFs)). However, Caspase 8 is also central to cell death induced by chemotherapeutic agents in a number of cancer cell types (Kim *et al*, 2001). Moreover, Caspase 8 is silenced by methylation in several other classes of cancer (Fulda, 2009). Similarly, *PYCARD* (also known as ASC/TMS1) is repressed by methylation in a range of tumours and has been implicated in several antitumour activities (Kim *et al*, 2001; McConnell and Vertino, 2004). In particular, *PYCARD* not only interacts with Caspase 8, but also can induce Caspase 8-dependent apoptosis (Masumoto *et al*, 2003), and it has also been implicated in apoptosis induced by a wide range of chemotherapeutic agents (Masumoto *et al*, 1999).

It is striking that among the tumour necrosis factor superfamily members and their receptors, six of the seven members represented on the arrays were selectively methylated in YSTs. These were *TNFSF8* and *TNFSF10* (the TRAIL ligand), and the TNFSF receptors, *TNFRSF10A*, *10C* and *10D* (three of the four death receptors for the TRAIL ligand) and *TNFRSF1B* (one of the other three death receptors acting via Caspase 8) (Guicciardi and Gores, 2009). Our data therefore provide a strong indication that methylation of genes associated with Caspase 8-dependent cell death might explain the relative resistance of YSTs to therapy-induced cell death (Fujimaki, 2009; Fulda, 2009). Therapeutic trials to activate *CASP8* that had been silenced by methylation have already taken place for other tumour types with some promising results (reviewed by Fulda, 2009).

### Relationship between the methylation status of germinomas and YSTs

Our data are consistent with a model in which the initiation of GCTs, such as germinomas, does not involve major changes in the methylation of gene regulatory elements, but subsequent methylation changes are then associated with the transition to a YST-like phenotype. Whether these changes are the cause or the result of this alteration in tumour phenotype is yet to be determined. This stepwise model may explain the occurrence of GCTs containing a mixture of seminoma and YST components (Looijenga, 2009) and the rare examples of seminomatous tumours that recur as a YST after therapy (Wong *et al*, 2010). Indeed, a recent report described a brain tumour that appeared to transform from a germinoma to a non-germinoma phenotype, even before treatment (Wong *et al*, 2010). A model in which all types of adult testicular GCTs, including seminomatous and non-seminomatous tumours, arise from a common carcinoma *in situ* (CIS) precursor lesion was proposed by Skakkebaek *et al* (1987).

### Mechanism of YST methylator phenotype

The substantial difference in methylation between germinomas and most YSTs suggests that a fundamental mechanism has been disrupted. Although it remains to be experimentally tested, our observation that *DNMT3B* is expressed at significantly higher levels in YSTs than in germinomas provides a possible explanation for this difference and, as YSTs exhibit a more aggressive natural history than germinomas, a target for potential therapy. Consistent with this suggestion, we found that 11 of about 70 genes recently identified as targets for DNMT3B1 (Choi *et al*, 2011) are among the genes methylated in our cohort of YSTs, whereas only two of the DNMT3A1-specific targets were methylated (all were present on our arrays). In addition, 23 of the genes that are targeted by both DNMTs were also methylated in the YST samples.

Interestingly, a recent study comparing microRNA expression profiles between paediatric YSTs and germinomas identified significant upregulation of all members of the miR-29 family (miR-29a, miR-29b and miR-29c) in germinomas (*P*=0.0001) (Murray *et al*, 2010), which correlates with the level of expression of *DNMT3A* and *DNMT3B* in the two subtypes of GCT. MicroRNA-29b has been shown to target and knock down expression of both *DNMT3A* and *DNMT3B* in cancer cells, with a consequent decrease in genome-wide methylation (Fabbri *et al*, 2007; Garzon *et al*, 2009). These observations therefore suggest a mechanism whereby relatively low levels of the miR-29 family members in YSTs may result in de-repression of *DNMT3B*, allowing the methylator phenotype to occur in this GCT subtype.

In conclusion, our data indicate that the methylator phenotype is a feature of the YSTs irrespective of anatomical location, patient age or sex. The gene targets of this methylation provide candidates for further analysis as potential tumour suppressors, especially components of Caspase 8-dependent apoptosis. Most importantly, our initial analysis suggests that the methylator phenotype, which is associated with the more aggressive subtype of tumour, the YST, is also associated with increased expression of *DNMT3B*. It will now be important to determine directly whether it is *DNMT3B* itself or another factor(s) that is the cause of the altered methylation.

## Figures and Tables

**Table 1:** Genes hypermethylated in more than 80% of YST samples analysed by GoldenGate array

Genes hypermethylated in more than 80% of YST samples, and in <25% of germinoma samples in the methylation array
***APC**, ASCL2, BDNF, CCNA1, CD2, CYP1B1, ESR1, HCK, HFE, HLA-F, **HOXA9**, HPN, HS3ST2, IGF2AS, IRAK3, IRF5, KLK10, LAT, NPY, NTSR1, OSM, PDGFRB, **PYCARD**, RASSF1, RIPK3, SCGB3A1, SLC22A3, STAT5A, TAL1, TNFSF8, VAV1, WNT1, WNT10B*

Abbreviations: YST=yolk sac tum.

Genes in bold were hypermethylated in all YSTs exhibiting the ‘methylator’ phenotype.

**Table 2:** List of genes that have previously been implicated as tumour suppressors among the 85 genes hypermethylated in YSTs

**Genes**	**Tumour type**	**Reference**
*RASSF1*	Testicular germ cell tumour; nasopharyngeal carcinoma	Honorio *et al* (2003); Koul *et al* (2004); Lind *et al* (2006); Wang *et al* (2009)
*HOXA9*	Breast cancer, testicular cancer	Lind *et al* (2006); Gilbert *et al* (2010)
*SCGB3A1*	Breast cell lines; mouse transformed Clara cells; testicular cancer	Krop *et al* (2005); Lind *et al* (2006); Tomita *et al* (2009)
*ESR1*	Squamous cell carcinoma	Zhai *et al* (2010)
*SLC22A3*	Prostate cancer	Tomlins *et al* (2007)
*PYCARD/ASC/TMS*	Leukemia-derived cell lines; colon adenocarcinoma and stomach cancer cell lines	Calvanese *et al* (2008); Mhyre *et al* (2009); Motani *et al* (2010)
*WNT2*	Colorectal cancer	Shi *et al* (2007)
*HIC1*	Paediatric neoplasm; head and neck squamous cell carcinoma	Rathi *et al* (2003); Koul *et al* (2004); Brieger *et al* (2010)
*APC*	Testicular germ cell tumour; invasive ductal carcinoma	Honorio *et al* (2003); Cho *et al* (2010)
*IRF5*	Gastric cancer	Yamashita *et al* (2010)
*SLIT2*	Lung cancer; glioma cell	Tseng *et al* (2010); Yiin *et al* (2009)
*TFAP2C*	Human extravillious throphoblast cell; breast adenocarcinoma	Li *et al* (2006); Kotani *et al* (2009)
*SLC5A8*	Head and neck squamous cell carcinoma	Bennett *et al* (2009)
*CASP8*	Neuroblastoma	Hoebeeck *et al* (2009)
*FES*	Mouse mast cells; colorectal Cancer	Shaffer and Smithgall (2009); Voisset *et al* (2010)
*WT1*	Ovarian clear–cell adenocarcinoma; gastric, lung, fibrosarcoma, glioblastoma	Kaneuchi *et al* (2005); Tatsumi *et al* (2008)
*CDH13*	Pituitary adenomas	Qian *et al* (2007); Andreeva and Kutuzov (2010)
*KLK10*	Non-small-cell lung cancer	Zhang *et al* (2010)
*LTB4R*	Colon cancer	Ihara *et al* (2007)
*THY1*	Nasopharyngeal carcinoma	Lung *et al* (2010)
*TNFRSF10C*	Prostate carcinoma	Hornstein *et al* (2008)
*COL1A2*	Colorectal cancer	Sengupta *et al* (2003)
*S100A4*	Pancreatic cancer cell lines	Tabata *et al* (2009)
*DCC*	Colon cancer cells	Rodrigues *et al* (2007)
*PTPRO*	Lung cancer	Motiwala *et al* (2004)
*TPEF*	Colon, bladder, prostate cancer	Liang *et al* (2010)

Abbreviation: YST=yolk sac tum.

Additional references in Supplementary information.

**Table 3:** Comparison between methylation status and expression levels for genes in which these show significant correlation

			**Delta beta-value**				**% Tumour methylated**			
			**1st cohort**		**2nd cohort**		**1st cohort**		**2nd cohort**	
**Genes**	**Ave. LODs**	**Tumour expression**	**Germ**	**YST**	**Germ**	**YST**	**Germ**	**YST**	**Germ**	**YST**
*TFAP2C*	28.71057	Germinoma	0.02692	0.50441	0.03356	0.38462	0	88	0	64
*PYCARD*	11.98783	Germinoma	0.02194	0.55576	0.06177	0.45505	0	100	0	91
*HDAC9*	9.563456	Germinoma	0.03521	0.31761	0.045	0.38154	0	38	0	55
*ETV1*	8.929844	Germinoma	0.04711	0.28954	0.17621	0.25473	0	38	11	36
*CD2*	8.079104	Germinoma	0.10937	0.52285	0.04331	0.41915	29	100	22	91
*CASP8*	7.686273	Germinoma	0.11408	0.41122	0.11146	0.40555	0	75	11	82
*HPN*	−7.431153	YST	0.14285	0.56466	0.16644	0.39917	29	100	22	82
*EVI2A*	4.376357	Germinoma	0.09123	0.38608	0.10608	0.3332	0	88	11	73
*HLA-F*	4.280225	Germinoma	0.0023	0.71691	0.01473	0.73472	0	100	0	73

Abbreviations: GCT=germ cell tumours; LOD=logarithm of odds; YST=yolk sac tum.

aveLODS represents the degree to which these genes are expressed at higher levels in germinomas than in YSTs (from Palmer *et al* (2008)). Methylation status is shown as the delta beta-value for difference in methylation between germinomas and YSTs in the two cohorts of GCTs analysed, and as a percentage of tumours in which the gene was significantly hypermethylated.
