# Supplementary material for: Methylator phenotype of malignant germ cell tumours in children identifies strong candidates for chemotherapy resistance
Source: Br J Cancer. 2011 Jun 28;105(4):575–85. doi: 10.1038/bjc.2011.218 (PMC3170957; doi:10.1038/bjc.2011.218)
Supplement: Supplementary Table S1 [file bjc2011218x1.doc]

Supplementary Table S1

| Histology | Site | Age | LINE1 Assay | Methylation Microarray |
| --- | --- | --- | --- | --- |
|  |  |  | % methylation | Phenotype |
| Germinoma | Ovary | 13 | N/A | N2 |
| Germinoma | Ovary | 15 | N/A | N2 |
| Germinoma | Ovary | 12 | N/A | N2 |
| Germinoma | Ovary | 9 | N/A | N2 |
| Germinoma | Ovary | 14 | 27 | N1 |
| Germinoma | Ovary | 11 | N/A | N/A |
| Germinoma | Ovary | 12 | 21 | N1 |
| Germinoma | Ovary | 13 | N/A | N2 |
| Germinoma | Ovary | 8 | 35 | N2 |
| Germinoma | Ovary | 12 | 14 | N/A |
| Germinoma | Ovary | 3 | N/A | N2 |
| Germinoma | Testis | 25 | 62 | N1 |
| Germinoma | Testis | 35 | N/A | N2 |
| Germinoma | Testis | 9 | 36 | N/A |
| Germinoma | Testis | 15 | N/A | N2 |
| Germinoma | Brain | 16 | 39 | N1 |
| Germinoma | Brain | 11 | 32 | N1 |
| Germinoma | Brain | 12 | 35 | N1 |
| Germinoma | Brain | 16 | 27 | N1 |
| YST | Ovary | 12 | 37 | N2 |
| YST | Ovary | 12 | 20 | M2 |
| YST | Ovary | 10 | N/A | M2 |
| YST | Ovary | 5 | 53 | M2 |
| YST | Ovary | 12 | 42 | M1 |
| YST | Ovary | 12 | 34 | M1 |
| YST | Ovary | 1 | N/A | M2 |
| YST | Testis | 1 | 44 | M1 |
| YST | Testis | 1 | 44 | M1 |
| YST | Testis | 1 | 53 | N/A |
| YST | Testis | 1 | 27 | M2 |
| YST | Testis | 1 | 54 | M2 |
| YST | Testis | 2 | 18 | N/A |
| YST | Testis | 17 | N/A | M2 |
| YST | Testis | N/A | N/A | M2 |
| YST | Brain | 12 | 32 | N/A |
| YST | Brain | 3 | 48 | M1 |
| YST | SCT | 16 | 73 | M2 |
| YST | SCT | 2 | 18 | M2 |
| YST | SCT | 2 | 83 | N/A |
| YST | SCT | 2 | 20 | M1 |
| YST | SCT | 2 | 37 | M1 |
| YST | Abdo | 12 | 49 | N/A |
| YST | Abdo | 0 | 57 | M1 |
| Control | Ovary | 0 | 34 | N/A |
| Control | Ovary | 0 | N/A | N/A |
| Control | Ovary | 0 | 83 | N/A |
| Control | Ovary | 0 | 59 | N/A |
| Control | Ovary | 43 | 67 | N/A |
| Control | Ovary | 7 | 73 | N/A |
| Control | Testis | 9 | 74 | N/A |
| Control | Testis | 0 | 80 | N/A |
| Control | Testis | 0 | 14 | N/A |
| Control | Fetal Yolk Sac | 0 | N/A | N/A |
| Control | Fetal Yolk Sac | 0 | 76 | N/A |
| Control | Fetal Yolk Sac | 0 | 62 | N/A |
| Control | Fetal Yolk Sac | 0 | 73 | N/A |
| Control | Buccal | 30 | 76 | N1&2 |
| Control | Buccal | 20 | 73 | N1&2 |
| Control | Buccal | 20 | 71 | N1&2 |
| Control | Blood | 1 | 73 | N1&2 |
| Control | Blood | 1 | 74 | N1&2 |
| Control | Blood | 2 | 73 | N1&2 |

List of tumours analysed in this study. YST – yolk sac tumour. Final two columns show percentage methylation of LINE-1 element, and overall methylation as determined by array analyses. N - non-methylator phenotype, M - methylator phenotype, Number (1 or 2) refers to whether the sample was in the first or second tumour sample cohort analysed.
