# Supplementary material for: Methylator phenotype of malignant germ cell tumours in children identifies strong candidates for chemotherapy resistance
Source: Br J Cancer. 2011 Jun 28;105(4):575–85. doi: 10.1038/bjc.2011.218 (PMC3170957; doi:10.1038/bjc.2011.218)
Supplement: Supplementary Table S2 Legend [file bjc2011218x3.doc]

**Supplementary Table S2**

XL file of raw DNA methylation data from Golden Gate methylation array analyses. Column A shows gene descriptors for each tag. Sheet ‘Raw data 1’ shows cohort 1 (columns B-U) and sheet ‘Raw data 2’ shows Cohort 2 (columns B-W). Germinomas (yellow header), YSTs (purple header) or controls (grey header). Columns V-X (cohort 1) and AC-AD (cohort 2) show average values for each class of sample.
