# Supplementary material for: Methylator phenotype of malignant germ cell tumours in children identifies strong candidates for chemotherapy resistance
Source: Br J Cancer. 2011 Jun 28;105(4):575–85. doi: 10.1038/bjc.2011.218 (PMC3170957; doi:10.1038/bjc.2011.218)
Supplement: Supplementary Table S3 [file bjc2011218x4.doc]

**Table S3 DNA primers used in this study.**

| **Primer** | **Sequence** | **Product Size (bp)** |
| --- | --- | --- |
| **1) LINE-1 Assay** | | |
| **Forward (FAM-labelled)** | **TTGAGTTGTGGTGGGTTTTATTTTAG** | **421** |
| **Reverse** | **TCATCTCACTAAAAAATACCAAACA** |
| **2) PYCARD pyrosequencing** | | |
| **Forward** | **TTAGTTGTTATGATTTTAAGATTT** | **294** |
| **Reverse (biotin-labelled)** | **CCAAACCTCTAAATTAAAACC** |
| **Sequencing** | **TTGTTATGATTTTAAGATTTTA** | **100** |
| **3) RT-PCR** | | |
| **i) DNMT3B** | | |
| **Forward** | **CGACAAGAGGGACATCTCACG** | **68** |
| **Reverse** | **CAGAAACTTTGATGGCATCAATCA** |
| **ii) ezh2** | | |
| **Forward** | **TAGGGAAGCAGGGACTGAAA** | **354** |
| **Reverse** | **CACAACCGGTGTTTCCTCTT** |
| **iii) Suz12** | | |
| **Forward** | **TGCAGTTCACTCTTCGTTGG** | **269** |
| **Reverse** | **TGCTTCAGTTTGTTGCCTTG** |
| **iv) Beta-actin** | | |
| **Forward** | **CTGGCACCCAGCACAATG** | **92** |
| **Reverse** | **GGACAGCGAGGCCAGGAT** |
| **v) 18S** | | |
| **Forward** | **CGCCGCTAGAGGTGAAATTCT** | **102** |
| **Reverse** | **CGAACCTCCGACTTTCGTTCT** |
