# Supplementary material for: Methylator phenotype of malignant germ cell tumours in children identifies strong candidates for chemotherapy resistance
Source: Br J Cancer. 2011 Jun 28;105(4):575–85. doi: 10.1038/bjc.2011.218 (PMC3170957; doi:10.1038/bjc.2011.218)
Supplement: Supplementary Information [file bjc2011218x5.doc]

**Supplementary References for Table 2**

1. Gilbert PM, Mouw JK, Unger MA, et al. HOXA9 regulates BRCA1 expression to modulate human breast tumor phenotype. J Clin Invest 2010; 120: 1535-50.

2. Tomita T, Yamada A, Miyakoshi M, et al. Oncostatin M regulates secretoglobin 3A1 and 3A2 expression in a bidirectional manner. Am J Respir Cell Mol Biol 2009; 40: 620-30.

3. Zhai Y, Bommer GT, Feng Y, Wiese AB, Fearon ER, Cho KR. Loss of estrogen receptor 1 enhances cervical cancer invasion. Am J Pathol 2010; 177: 884-95.

4. Krop I, Parker MT, Bloushtain-Qimron N, et al. HIN-1, an inhibitor of cell growth, invasion, and AKT activation. Cancer Res 2005; 65: 9659-69.

5. Tomlins SA, Mehra R, Rhodes DR, et al. Integrative molecular concept modeling of prostate cancer progression. Nat Genet 2007; 39: 41-51.

6. Calvanese V, Horrillo A, Hmadcha A, et al. Cancer genes hypermethylated in human embryonic stem cells. PLoS One 2008; 3: e3294.

7. Mhyre AJ, Marcondes AM, Spaulding EY, Deeg HJ. Stroma-dependent apoptosis in clonal hematopoietic precursors correlates with expression of PYCARD. Blood 2009; 113: 649-58.

8. Motani K, Kawase K, Imamura R, Kinoshita T, Kushiyama H, Suda T. Activation of ASC induces apoptosis or necrosis, depending on the cell type, and causes tumor eradication. Cancer Sci 2010; 101: 1822-7.

9. Shi Y, He B, Kuchenbecker KM, et al. Inhibition of Wnt-2 and galectin-3 synergistically destabilizes beta-catenin and induces apoptosis in human colorectal cancer cells. Int J Cancer 2007; 121: 1175-81.

10. Yamashita M, Toyota M, Suzuki H, et al. DNA methylation of interferon regulatory factors in gastric cancer and noncancerous gastric mucosae. Cancer Sci 2010; 101: 1708-16.

11. Yiin JJ, Hu B, Jarzynka MJ, et al. Slit2 inhibits glioma cell invasion in the brain by suppression of Cdc42 activity. Neuro Oncol 2009; 11: 779-89.

12. Tseng RC, Lee SH, Hsu HS, et al. SLIT2 attenuation during lung cancer progression deregulates beta-catenin and E-cadherin and associates with poor prognosis. Cancer Res 2010; 70: 543-51.

13. Kotani T, Iwase A, Ino K, et al. Activator protein-2 impairs the invasion of a human extravillous trophoblast cell line. Endocrinology 2009; 150: 4376-85.

14. Li H, Goswami PC, Domann FE. AP-2gamma induces p21 expression, arrests cell cycle, and inhibits the tumor growth of human carcinoma cells. Neoplasia 2006; 8: 568-77.

15. Bennett KL, Romigh T, Eng C. Disruption of transforming growth factor-beta signaling by five frequently methylated genes leads to head and neck squamous cell carcinoma pathogenesis. Cancer Res 2009; 69: 9301-5.

16. Voisset E, Lopez S, Chaix A, et al. FES kinase participates in KIT-ligand induced chemotaxis. Biochem Biophys Res Commun 2010; 393: 174-8.

17. Shaffer JM, Smithgall TE. Promoter methylation blocks FES protein-tyrosine kinase gene expression in colorectal cancer. Genes Chromosomes Cancer 2009; 48: 272-84.

18. Kaneuchi M, Sasaki M, Tanaka Y, et al. WT1 and WT1-AS genes are inactivated by promoter methylation in ovarian clear cell adenocarcinoma. Cancer 2005; 104: 1924-30.

19. Tatsumi N, Oji Y, Tsuji N, et al. Wilms' tumor gene WT1-shRNA as a potent apoptosis-inducing agent for solid tumors. Int J Oncol 2008; 32: 701-11.

20. Andreeva AV, Kutuzov MA. Cadherin 13 in cancer. Genes Chromosomes Cancer 2010; 49: 775-90.

21. Qian ZR, Sano T, Yoshimoto K, et al. Tumor-specific downregulation and methylation of the CDH13 (H-cadherin) and CDH1 (E-cadherin) genes correlate with aggressiveness of human pituitary adenomas. Mod Pathol 2007; 20: 1269-77.

22. Zhang Y, Song H, Miao Y, Wang R, Chen L. Frequent transcriptional inactivation of Kallikrein 10 gene by CpG island hypermethylation in non-small cell lung cancer. Cancer Sci 2010; 101: 934-40.

23. Ihara A, Wada K, Yoneda M, Fujisawa N, Takahashi H, Nakajima A. Blockade of leukotriene B4 signaling pathway induces apoptosis and suppresses cell proliferation in colon cancer. J Pharmacol Sci 2007; 103: 24-32.

24. Lung HL, Cheung AK, Cheng Y, et al. Functional characterization of THY1 as a tumor suppressor gene with antiinvasive activity in nasopharyngeal carcinoma. Int J Cancer 2010; 127: 304-12.

25. Hornstein M, Hoffmann MJ, Alexa A, et al. Protein phosphatase and TRAIL receptor genes as new candidate tumor genes on chromosome 8p in prostate cancer. Cancer Genomics Proteomics 2008; 5: 123-36.

26. Tabata T, Tsukamoto N, Fooladi AA, et al. RNA interference targeting against S100A4 suppresses cell growth and motility and induces apoptosis in human pancreatic cancer cells. Biochem Biophys Res Commun 2009; 390: 475-80.

27. Motiwala T, Kutay H, Ghoshal K, et al. Protein tyrosine phosphatase receptor-type O (PTPRO) exhibits characteristics of a candidate tumor suppressor in human lung cancer. Proc Natl Acad Sci U S A 2004; 101: 13844-9.

28. Liang G, Robertson KD, Talmadge C, Sumegi J, Jones PA. The gene for a novel transmembrane protein containing epidermal growth factor and follistatin domains is frequently hypermethylated in human tumor cells. Cancer Res 2000; 60: 4907-12.

29. Cho YH, Yazici H, Wu HC, et al. Aberrant promoter hypermethylation and genomic hypomethylation in tumor, adjacent normal tissues and blood from breast cancer patients. Anticancer Res 2010; 30: 2489-96.

30. Sengupta PK, Smith EM, Kim K, Murnane MJ, Smith BD. DNA hypermethylation near the transcription start site of collagen alpha2(I) gene occurs in both cancer cell lines and primary colorectal cancers. Cancer Res 2003; 63: 1789-97.

31. Wang T, Liu H, Chen Y, Liu W, Yu J, Wu G. Methylation associated inactivation of RASSF1A and its synergistic effect with activated K-Ras in nasopharyngeal carcinoma. J Exp Clin Cancer Res 2009; 28: 160.

32. Rodrigues S, De Wever O, Bruyneel E, Rooney RJ, Gespach C. Opposing roles of netrin-1 and the dependence receptor DCC in cancer cell invasion, tumor growth and metastasis. Oncogene 2007; 26: 5615-25.

33. Hoebeeck J, Michels E, Pattyn F, et al. Aberrant methylation of candidate tumor suppressor genes in neuroblastoma. Cancer Lett 2009; 273: 336-46.
